# Expression of Concern: Relationship between microRNA-27a and efficacy of neoadjuvant chemotherapy in gastric cancer and mechanism in gastric cancer cell growth and metastasis

**DOI:** 10.1042/BSR-20181175_EOC

**Published:** 2020-07-29

**Keywords:** Cell growth, Efficacy, Gastric cancer, Metastasis, MicroRNA-27a, Neoadjuvant chemotherapy

The Editorial Office has been made aware of potential issues surrounding the scientific validity of this paper, hence has issued an expression of concern to notify readers whilst the Editorial Office investigates.

